# The complete mitochondrial genome of the Malagasy clouded mother-of-pearl butterfly *Protogoniomorpha ancardii duprei* (Insecta: Lepidoptera: Nymphalidae)

**DOI:** 10.1080/23802359.2020.1810156

**Published:** 2020-08-31

**Authors:** Melanie M. L. Lalonde, Jeffrey M. Marcus

**Affiliations:** Department of Biological Sciences, University of Manitoba, Winnipeg, Canada

**Keywords:** Illumina sequencing, masquerade mimicry, Nymphalinae, Junoniini, mitogenomics

## Abstract

The Malagasy clouded mother-of-pearl butterfly, *Protogoniomorpha ancardii duprei* (Nymphalidae), is the Madagascar subspecies of a widespread sub-Saharan leaf-mimic. Genome skimming allowed the assembly of the complete *P. ancardii duprei* circular mitogenome (15,220 bp) consisting of 80% AT nucleotides, 13 protein-coding genes, 22 tRNAs, two rRNAs, and a control region in typical butterfly gene order. *Protogoniomorpha ancardii duprei COX1* has a CGA start codon while *COX1, COX2, CYTB, NAD1*, and *NAD4* exhibit partial stop codons completed by 3′ A residues added to the mRNA. Phylogenetic reconstruction places *Protogoniomorpha* as sister to genus *Yoma* within monophyletic tribe Junoniini.

The Malagasy clouded mother-of-pearl butterfly *Protogoniomorpha ancardii duprei* Vinson, 1863 (Nymphalidae) is an endemic leaf mimic (Suzuki et al. 2014) found in the forests and forest margins of Madagascar (Lees et al. 2003). This species is an example of masquerade mimicry (Skelhorn 2015) and was one of the several species that had been placed within genus *Salamis*, but were recently moved to genus *Protogoniomorpha* based on morphological and molecular analyses (Wahlberg et al. 2005; Suzuki et al. 2014). Here, we report the complete mitochondrial genome sequence of *P. ancardii duprei* from specimen Sana2015.1 that was collected in Andasibe, Madagascar (GPS 18.835S, 48.457E) in March 2015. It has been pinned, spread, and deposited in the Wallis Roughley Museum of Entomology, University of Manitoba (voucher WRME0507728).

DNA was prepared (McCullagh and Marcus 2015) and sequenced by Illumina NovaSeq6000 (San Diego, CA). The mitogenome of *P. ancardii duprei* (GenBank MT702382) was assembled by Geneious 10.1.2 from 10,826,141 paired 150 bp reads using a *Salamis anteva* (Lepidoptera: Nymphalidae) reference mitogenome (MH917707) (Lalonde and Marcus 2019). Annotation was in reference to *S. anteva* and *Precis andremiaja* (MH917706) (Lalonde and Marcus 2019) mitogenomes. The *P. ancardii duprei* nuclear rRNA repeat (GenBank MT702383) was also assembled and annotated using *S. anteva* (MH917709) and *P. andremiaja* (MH917708) reference sequences.

The *P. ancardii duprei* circular 15,220 bp mitogenome assembly was composed of 47,362 reads with nucleotide composition: 40.1% A, 12.3% C, 7.7% G, and 39.9% T. Gene composition and gene order in *P. ancardii duprei* matches currently known butterfly mitogenomes (McCullagh and Marcus 2015). The *P. ancardii duprei* mitochondrial protein-coding gene start codons include: ATG (ATP6, *COX2*, *COX3*, *NAD4*, *NAD4L*, *CYTB*, *NAD1*), ATT (*NAD2*, *NAD5*, *NAD6*), ATC (*ATP8*, *NAD3*), and CGA (*COX1*). This mitogenome contains four protein-coding genes (*COX1*, *COX2, NAD4, CYTB*) with single-nucleotide (T) stop codons, and one protein-coding gene (*NAD1*) with a two-nucleotide (TA) stop codon completed by post-transcriptional addition of 3′ A residues. The structures and locations of tRNAs were determined using ARWEN v.1.2 (Laslett and Canback 2008). All tRNAs have typical cloverleaf secondary structures except for trnS (AGN) where the dihydrouridine arm is replaced by a loop, while the mitochondrial rRNAs and control region are typical for Lepidoptera (McCullagh and Marcus 2015).

Phylogeny was reconstructed using complete mitogenomes from *P. ancardii duprei*, 28 additional mitogenomes from tribe Junoniini, and four outgroup species from other tribes in subfamily Nymphalinae (Peters and Marcus 2016, 2017; Lalonde and Marcus 2019; Living Prairie Mitogenomics Consortium 2020). Sequences were aligned in CLUSTAL Omega (Sievers et al. 2011) and analyzed by parsimony and maximum likelihood (model selected by jModeltest 2.1.7 (Darriba et al. 2012) and likelihood ratio test (Huelsenbeck and Rannala 1997)) in PAUP* 4.0b8/4.0d78 (Swofford 2002) (Figure 1). Phylogenetic analysis places *P. ancardii duprei* as sister to *Yoma sabina* which is contrary to traditional morphology-based taxonomy (which placed *P. ancardii* in genus *Salamis* (Vinson 1863)) but is in agreement with previous molecular phylogenetic reconstructions (Wahlberg et al. 2005; Kodandaramaiah and Wahlberg 2007).

**Figure 1.**
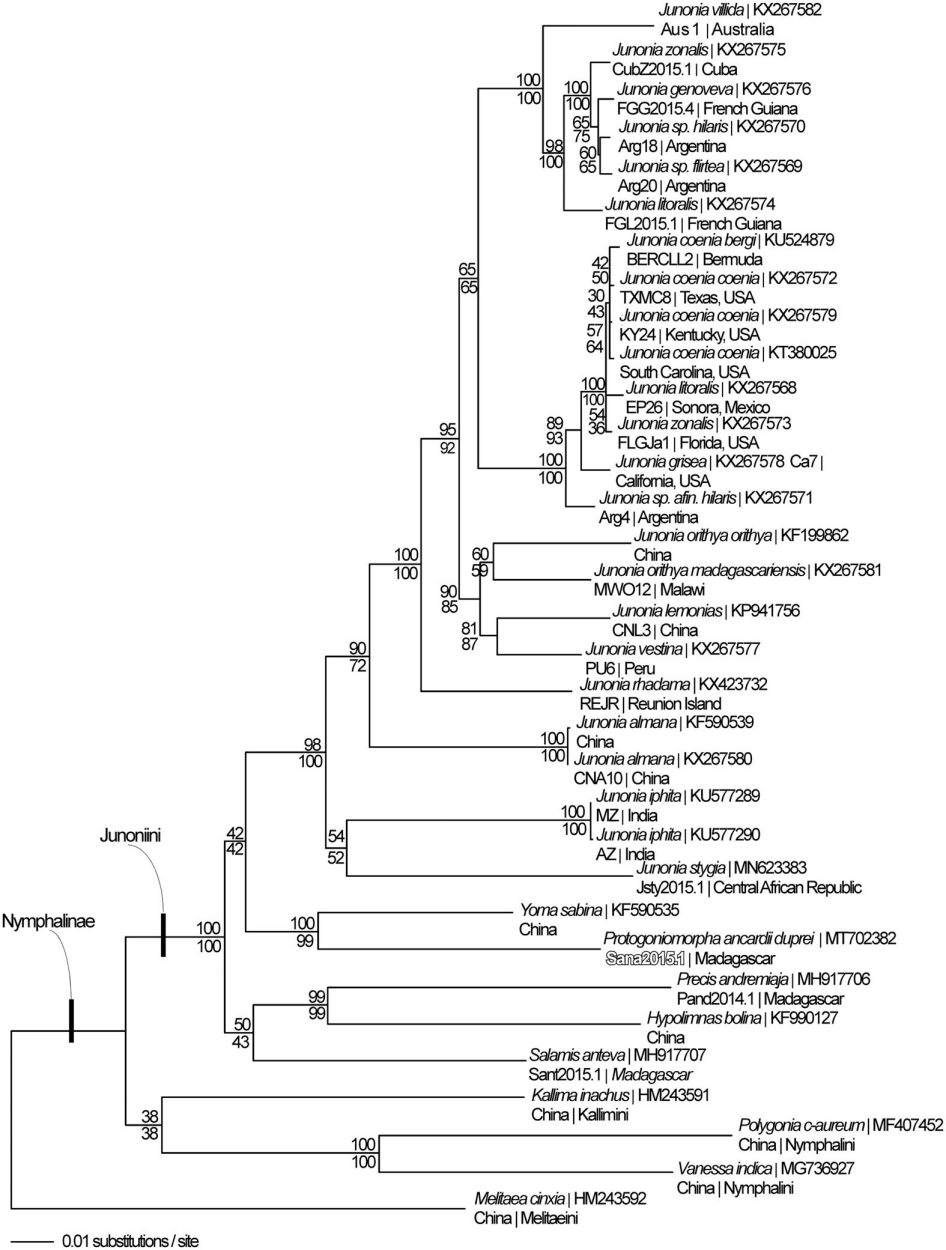
Maximum likelihood phylogeny (GTR + G model, likelihood score (84716.13)) of *Protogoniomorpha ancardii duprei*, 28 additional mitogenomes from tribe Junoniini, and 4 outgroup species from other tribes in subfamily Nymphalinae based on 1 million random addition heuristic search replicates (with tree bisection and reconnection). One million maximum parsimony heuristic search replicates produced 8 trees (parsimony score 13,373 steps) which differ from one another only by the arrangement of *Junonia coenia* mitogenomes and one of which has an identical tree topology to the maximum likelihood tree depicted here. Numbers above each node are maximum likelihood bootstrap values and numbers below each node are maximum parsimony bootstrap values (each from 1 million random fast addition search replicates).

## Data Availability

The data that support the findings of this study are openly available in GenBank of NCBI at https://www.ncbi.nlm.nih.gov, reference numbers MT702382 and MT702383.
